# Proteomic Markers of Non-functional Overreaching During the Race Across America (RAAM): A Case Study

**DOI:** 10.3389/fphys.2019.01410

**Published:** 2019-11-15

**Authors:** Edward K. Merritt, David C. Nieman, Brian R. Toone, Arnoud Groen, Artyom Pugachev

**Affiliations:** ^1^Department of Kinesiology, Southwestern University, Georgetown, TX, United States; ^2^North Carolina Research Campus, Appalachian State University, Kannapolis, NC, United States; ^3^Department of Mathematics and Computer Science, Samford University, Homewood, AL, United States; ^4^ProteiQ Biosciences GmbH, Berlin, Germany

**Keywords:** cycling, overreach, overtraining syndrome, ultraendurance, RAAM, proteomics

## Abstract

In a previous study, proteomics procedures identified blood proteins as potential overreaching and overtraining biomarkers, and a targeted proteomics panel of 21 proteins was developed.

**Purpose:**

To measure targeted blood protein changes in an ultraendurance cyclist competing in RAAM.

**Methods:**

The athlete underwent testing 4-week pre-RAAM and 4-day post-RAAM to determine body composition and aerobic capacity. During RAAM training distress score (TDS) and body mass were measured daily. Power output and heart rate (HR) were measured during cycling. Blood sampling for proteomic analysis occurred 4 weeks, 24, and 2 h before the start, twice per day of the race, and after 1 and 4 days recovery.

**Results:**

The athlete completed the 4941 km race in 10.1 days at a speed of 24.5 km/h with 20 total hours of sleep. TDS was very low, 1, pre-RAAM and increased to very high, 47, at the finish. Post-RAAM maximal aerobic capacity and HR were 6.3 and 5.7% lower (61.6 vs. 57.5 mL.kg^–1^.min^–1^ and 192 bpm vs. 181 bpm). Body composition did not change. The change in blood proteins was calculated using pre-race samples and samples collected on days 8, 9, and recovery day 1. The blood proteins with the largest increase were complement component C7 (359%), complement C4-B (231%), serum amyloid A-4 protein (210%), inter-alpha-trypsin inhibitor heavy chain H4 (191%), and alpha-1-antitrypsin (188%).

**Conclusion:**

The RAAM athlete exhibited non-functional overreaching symptoms including increased training distress and decreased work capacity. Proteomic analysis indicated large increases for immune-related proteins involved with complement activation and the acute phase response, which could be useful biomarkers for non-functional overreaching.

## Introduction

Athletes, military personnel, and others involved in strenuous physical training must match the demands of the activities with adequate recovery in order to maximize performance (Meeusen et al., 2013; Soligard et al., 2016). The balance between the physiologic stress, overload, of training and the recovery process, which drives the physiologic adaptations, is crucial for high-level performance. Low levels of overload are not sufficiently strenuous to cause physiological adaptations benefiting performance, but even high levels of overload followed by long recovery periods may lead to insufficient performance improvements. To maximize performance adaptations, athletes routinely employ a concept known as functional overreaching. These training blocks focus on the overload training principle and precede a recovery period. During and immediately after the training block, a temporary performance deficit occurs but resolves over a short period of time, ultimately leading to an improvement in performance in the days and weeks afterward (Aubry et al., 2014). Conversely, a concept known as non-functional overreaching (NFOR) occurs if recovery is not sufficient given the volume and/or intensity of training (Meeusen et al., 2013). NFOR contributes to a long-term performance deficit, known as overtraining syndrome (OTS), which lasts for weeks or even months. The body is unable to adapt optimally to improve performance. Those hoping to maximize performance must be careful not to push beyond the line between the correct amount of training/recovery and too much training/not enough recovery. Unfortunately, the transition between this functional and non-functional overreaching is difficult to determine as there are few if any objective indicators to define the transition. In fact subjective measures are the most reliable indicators of overreaching, superior to objective physiologic measures such as plasma hormones and cytokines, energy homeostasis, and exercise workload monitoring (Saw et al., 2016). Mastering the balance of overload and recovery can be enhanced with better knowledge of the objective physiological responses indicative of the transition to NFOR.

Ultra-endurance athletes exercise nearly continuously for days or weeks with little or no recovery and provide an opportunity to study overreaching and potentially the transition from functional overreaching to NFOR. During these events, athletes experience the classic symptoms of overreaching including inflammation, muscle damage, fatigue, and decreased innate immune function (Nieman et al., 2003, 2005; Kupchak et al., 2014; Keohane et al., 2019; Nieman and Wentz, 2019). The Race Across America (RAAM), a 4800+ km cycling race during which competitors cross the United States on a designated route as fast as possible, provides a unique physiologic stimulus to further delineate normal, acute training biomarkers of functional overreaching from NFOR and OTS (Knechtle et al., 2005; Hulton et al., 2010). The RAAM rules require that athletes cross the country in less than 13 days. The top competitors normally complete the 4800+ km route in 8–10 days, which is only possible by minimizing time off the bike and sleeping as little as possible (<2–3 hours/day). With 20+ h per day spent cycling, recovery is nearly non-existent. The extreme nature of the event provides a unique opportunity to monitor the physiologic changes that occur due to NFOR. Recent proteomic work identified likely biomarkers of NFOR and OTS (Nieman et al., 2018). The biomarkers identified were not upregulated acutely due to exercise itself, but were elevated in the recovery phase. The goal of this project was to confirm if these biomarkers were elevated and monitor their progression during RAAM, a known case of NFOR.

## Materials and Methods

Prior to RAAM, a competing athlete was identified. The athlete provided informed consent for testing and blood sampling before, during, and after RAAM. The Appalachian State University Institutional Review Board approved all procedures, which were in accordance with the Declaration of Helsinki.

### Pre-RAAM Testing

Four-weeks prior to RAAM start, the subject visited the laboratory for baseline measurements at least 4-h post-prandial. Following completion of a health-history questionnaire, the subject’s height and body mass were measured. Body composition was determined via dual energy x-ray absorptiometry (DXA; Discovery Hologic, Toronto, ON, Canada). A fingerprick blood sample was obtained from the subject at rest for proteomic analysis. Maximal oxygen consumption was determined by a standard maximal graded exercise test on a cycle ergometer (Lode Excalibur Sport, Groningen, Netherlands). The subject began at a workload of 150 W and every 2 min the workload was increased by 25 W. Exhaled respiratory gases were analyzed with the Cosmed CPET system (Rome, Italy).

The day before and 2-h prior to the race start, the subject’s body mass was determined and fingerprick blood samples were obtained. Two-hours prior to RAAM start the subject also completed a training distress scale (TDS) questionnaire, a brief self-report questionnaire, which asks questions about the athlete’s physical and mental well-being (Grove et al., 2014). Responses to each question are scored on a 0–4 point scale with higher numbers indicating more distress. Total TDS scores over 16 have been associated with performance deficits (Grove et al., 2014).

### RAAM Testing

During RAAM, power output (Quarq, SRAM, Chicago, IL, United States), cycling time, distance traveled, heart rate, global positioning system (GPS) data, and environmental conditions were monitored (Garmin 810, Olathe, KS, United States) continuously while the subject was on the bicycle. Time and duration of rest and sleep breaks were recorded by the GPS data and race crew. Fingerprick blood samples were obtained and body mass was determined twice per day during subject’s stops (with the exception of two missed sample collections on Day 6 pm and Day 8 pm). Samples were obtained 10–15 h after the previous collection in order to better track the trends of the data over time. Once per day during rest breaks (except Day 8), the TDS questions were read aloud to the subject and answers were recorded.

### Post-RAAM Testing

Body mass, fingerprick blood samples, and TDS answers were obtained after RAAM finish. Data was collected 2-h post-RAAM, 18-h post-RAAM, and 4-days post-RAAM. Body composition and maximal oxygen consumption measurements were repeated 4-days post-RAAM as done pre-RAAM.

### Dried Blood Spot Proteomic Analysis

Blood drops from fingerprick blood sampling were dried on standard blood spot cards (Whatman Protein Saver Cards, Sigma-Aldrich, St. Louis, MO, United States). Blood analysis was performed as previously described (Nieman et al., 2018). Proteins were resolubilized from the DBS and digested with trypsin before proteomics measurements (Multiple Reaction Monitoring) on an Agilent 6400 QqQ LC-MS/MS. Heavy isotope labeled synthetic peptide standards were spiked in for correct identification and normalization. Twenty one individual proteins were quantified (see Table 1 for descriptions of each). Data was analyzed using Skyline. Normalization of endogenous peptide values was done by correction on the median intensity of the heavy standards. The% change in blood proteins was calculated using the average of pre-race samples and 5 samples collected on days 8, 9, and the first day of recovery.

**TABLE 1:** Proteins included for analysis in the targeted proteomics panel prior to, during, and after completion of RAAM.

**UniProt protein**	**Protein name**	**Basic function**
P35542	Serum amyloid A-4 protein (SAA4)	Major acute phase reactant; cell chemotaxis
P05164	Myeloperoxidase (PERM)	Granulocyte microbicidal activity against wide range of pathogens; production of hypochlorous acid
P07360	Complement component C8 gamma chain (CO8)	Part of membrane attack complex that plays key role in immune response; forms pores in target cells
P0C0L5	Complement C4B (CO4B)	Non-enzymatic component C3, C5 convertases and thus essential for complement activation
P05155	Plasma protease C1 inhibitor (IC1)	Crucial role in regulation of complement activation.
Q14624	Inter-alpha-trypsin inhibitor heavy chain H4 (ITIH4)	Acute-phase protein involved in trauma inflammatory response
P19652	Alpha-1-acid glycoprotein 2 (A1AG2)	Transport protein; modulates immune function during the acute-phase reaction; inflammation
P10643	Complement component C7 (CO7)	Part of membrane attack complex that plays key role in immune response; forms pores in target cells
P02765	Alpha-2-HS-glycoprotein (FETUA)	Promotes endocytosis; part of acute-phase response; phagocytosis; bone mineral influence
P01834	Immunoglobulin kappa constant (IGKC)	Constant region of immunoglobulin heavy chains; complement activation; defense immune response; phagocytosis recognition and engulfment
P08185	Corticosteroid-binding globulin (CBG)	Major transport protein for glucocorticoids and progestins
P35754	Glutaredoxin-1 (GLRX1)	Glutathione activity; cell redox homeostasis
P00568	Adenylate kinase isoenzyme 1 (KAD1)	Role in cellular energy homeostasis; nucl. diphoph. kinase activity.
P01009	Alpha-1-antitrypsin (A1AT)	Inhibitor of serine proteases; primary target is elastase
P01011	Alpha-1-antichymotrypsin (AACT)	Inhibition of neutrophil cathepsin G and mast cell chymase
P08603	Complement factor H (CFAH)	Maintenance of a well-balanced immune response by modulating complement activation
P10599	Thioredoxin (THIO)	Participates in various redox reactions
P15531	Nucleoside diphosphate kinase A (NDKA)	Synthesis of nucleoside triphosphates other than ATP.; Required for neural development including neural patterning and cell fate determination.
P19827	Inter-alpha-trypsin inhibitor heavy chain H1 (ITIH1)	Involved in hyaluronan metabolic process
Q08380	Galectin-3-binding protein (LG3BP)	Host defense against viruses and tumor cells; promotion of integrin-mediated cell adhesion
Q9UQ80	Proliferation-associated protein 2G4 (PA2G4)	Growth regulation; ERBB3-regulated signal transduction pathway

## Results

The 40-year old male subject completed RAAM in just over 10 days finishing in the Top 5 overall. See Table 2A for performance statistics. On RAAM day 1 his TDS was 1 (very low distress), and this number reached 34 (high distress) by Day 2 after more than 1200 km of riding with less than 2 h of sleep. TDS gradually increased until Day 7 where it peaked at 47 (very high distress) after which the number stayed in the mid-40 s peaking again at 47 immediately after finishing RAAM on Day 10. The subject did not experience any symptoms of illness nor infection during the time course of the study. During the race, the subject rode near continuously for 26–28 h with short, interspersed rest/food/restroom breaks. On eight separate occasions, the subject stopped to sleep for more than 20 min, averaging 2.0 total hours of sleep/24 h period. Average cycling power output was 102.6 ± 8.9 Watts with the highest 24-h power averages occurring in the first and last 24 h (108 Watts vs. 120 Watts).

**TABLE 2:** **(A)** Performance Statistics during RAAM. **(B)** Pre- and Post-RAAM body composition and maximal aerobic capacity tests.

**(A) Race Across America Statistics**		**(B)**	**Pre-RAAM**	**Post-RAAM**
Distance	4941 km	Body Mass (kg)	67.9	68.0
Time	10.12 days	% Body fat	9.7	9.3
Average Moving Speed	24.5 km/h	Heart rate max (beats/min)	192	181
Average Moving Time	20.0 h/day	VO_2_ max (ml.kg^–1^.min^–1^)	61.6	57.5
Average Power	103 Watts	Power @ Aerobic Max (W)	350	325
Total Sleep	20.0 h			

Training distress score returned to near baseline levels 4-days post-RAAM (TDS = 2). No changes in body mass or composition occurred pre-RAAM to post-RAAM (Table 2B). Maximal aerobic capacity was 6.3% lower (VO_2_ max = 61.6 mL.kg^–1^.min^–1^ vs. 57.5 mL.kg^–1^.min^–1^) and maximal heart rate was 5.7% lower (192 vs. 181 beats/min) than pre-RAAM measures (Table 2B). The subject achieved maximal exertion during each test as evidenced by plateaus in oxygen consumption and heart rate with increased ergometer workload, a respiratory exchange ratio greater than 1.10, and reporting a maximal rating of perceived exertion.

**FIGURE 1:**
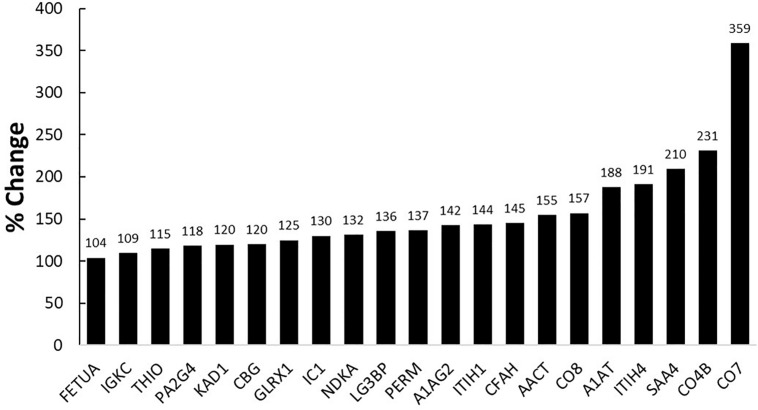
Percent change in targeted blood proteins during RAAM. A previous study (Nieman et al., 2018) determined that these proteins are not acutely upregulated following an exercise bout, potentially implicating them as strong candidates for NFOR monitoring in individuals.

**FIGURE 2:**
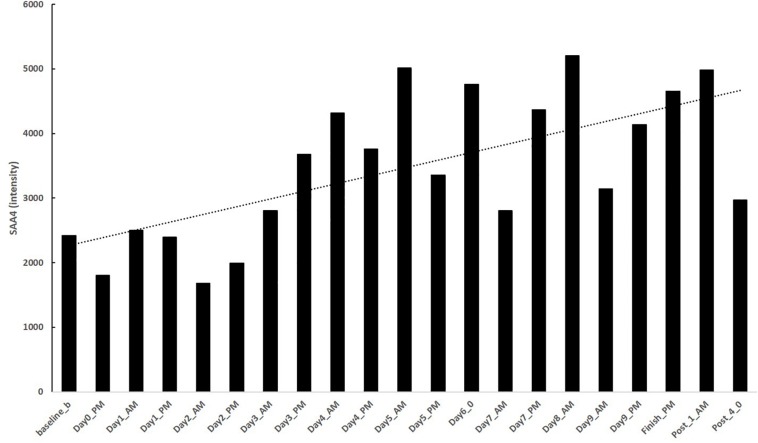
Intensity values for serum amyloid A4 (SAA4) before, during (morning and evening), and after RAAM competition.

Blood sample analysis indicated the largest serum protein increases during RAAM were immune-related proteins involved in complement activation and the acute phase response (Figure 1). A 359% increase in complement component C7 (CO7), a 231% increase in complement C4-B (CO4B), a 210% increase in serum amyloid A-4 protein (SAA4) (see Figure 2 for the time course of changes in SAA4), a 191% increase in Inter-α-trypsin inhibitor heavy chain H4 (ITIH4), and a 188% increase in α-1-antitrypsin (A1AT), occurred due to RAAM.

## Discussion

Utilizing the 4941 km RAAM completed in 10.12 days as a field-based case study of overreaching, the data collected confirm NFOR and identify several potential biomarkers of NFOR. Completing RAAM led to an extraordinary increase in TDS and a subsequent decrease in functional capacity (as evidence by lower VO_2_ peak and cycling power at maximal effort), both hallmarks of NFOR. The demanding nature of the race was evident in the subject’s continually increasing training distress. Increasing TDS values are associated with decreases in performance capability with scores as low as 16 related to decreased endurance performance (Grove et al., 2014). Prior to the RAAM start, the subject’s TDS was 1 indicating very low distress, but just over 48 h into the race, the TDS had increased dramatically to 34 and continued to increase until the finish with a TDS of 47. These far exceed scores expected in athletes experiencing performance deficits associated with NFOR. Four days after completing RAAM, even though the subject’s TDS returned to near baseline (*TDS* = 2), functional capacity was still impaired. Interestingly, previous work has indicated that subjective measures of an athlete’s well-being are better indicators of NFOR/OTS (Saw et al., 2016), however in this case-study, the subjective measures did not predict the lowered functional capacity 4-days post-RAAM.

The changes in training distress during RAAM were accompanied by a change in the proteomic profile of the subject’s blood (Figure 1), and this discussion will focus on the top five proteins with the largest increase. The five proteins that increased the most are involved in the acute phase innate immune response, but based on previous work (Nieman et al., 2018), are not involved in the acute exercise response. SAA4, A1AT, and CO4 have all previously been investigated in horses in various states of training distress (Bouwman et al., 2010; Cywinska et al., 2010; Scoppetta et al., 2012; Turło et al., 2015), but only limited data exist on their roles in training distress in humans.

Serum amyloid A has been proposed as a biomarker of value for monitoring intensified stress including overuse injury in racehorses (Turło et al., 2015). In humans, SAA was high 2 days after the finish of the 246 km ultraendurance running Spartathlon race, despite other acute markers returning to normal (Margeli et al., 2005; Balfoussia et al., 2014). In a functional overreaching study of runners and cyclists, SAA4 did not increase acutely post-exercise but only during the recovery phase (Nieman et al., 2018). Similar to these findings, our data indicate that the more than 200% increase in SAA4 at the end of RAAM and during recovery implicate SAA4 as a strong candidate for monitoring of NFOR.

Another proposed biomarker of value studied in racehorses in which we saw a nearly 200% increase during RAAM is A1AT. In horses, A1AT is increased with prolonged physical exertion and increases with intensified training similar to functional overreaching but not with standard training (Bouwman et al., 2010; Scoppetta et al., 2012). In humans, A1AT does not appear to increase solely due to single, stressful bouts of endurance exercise as A1AT levels were no different prior to and for 48-h after a half-marathon running race (Vassalle et al., 2018). However, A1AT was significantly higher after the accumulated stress of the 3-week cycling race, the Vuelta a España (Semple et al., 2006). Interestingly it was not higher after only 1 week of racing, when the accumulated stress on this group of professional endurance athletes is likely within the high end of their normal realm of training and racing volume. The increase in A1AT due to the accumulated stress of RAAM indicates that it is a good candidate as a marker for NFOR.

Our data indicated a 231% increase in CO4B due to RAAM. As with the other biomarkers, CO4 increases in racehorses with prolonged physical exertion (Scoppetta et al., 2012). Similarly, CO4 also increases in humans after 10 days of endurance cycling in professional cyclists at the Vuelta a España race (Semple et al., 2006). In a functional overreaching study, CO4B increased on the recovery days, but not acutely due to exercise (Nieman et al., 2018). As with SAA4, CO4B could be a strong signal for functional overreaching transitioning to NFOR.

The marker with the largest increase due to RAAM was CO7, an acute-phase protein that plays a key role in the immune and inflammatory responses (Morgan, 2016). Few studies have analyzed the role of CO7 with exercise, although it increased during recovery following functional overreaching in humans (Nieman et al., 2018). Similarly ITIH4, another acute-phase protein involved in the trauma inflammatory response, had a 191% increase due to RAAM, but also has limited data in exercise. ITIH4 also increased during recovery following functional overreaching in humans (Nieman et al., 2018). This is the first study to indicate that CO7 and ITIH4 increase during NFOR.

Important factors to note in this case-study are total energy input and output and sleep deprivation. Total energy input and output were well-matched as evidenced by the lack of change in body mass or composition over the course of RAAM. Given the duration of the event, the subject’s performance was approaching the limits of maximal sustained metabolic scope based off previous estimates (Thurber et al., 2019). Energy imbalance, independent of training, could lead to changes in many potential biomarkers of overreaching (Poitou et al., 2005; Reed et al., 2010). The energetic balance achieved by the subject during RAAM further strengthens the likelihood that the biomarkers measured here are due to NFOR and not energy deprivation. However, limited data are available on the potentially independent effects of sleep disruption combined with stressful exercise on these biomarkers. Obviously, due to the nature of RAAM, sleep could not be controlled, but this should be considered in future studies.

## Conclusion

Our results confirm that several of the previously hypothesized biomarkers of NFOR were upregulated due to RAAM. Due to the training distress and physiologic demands of completing RAAM, decrease in functional capacity post-RAAM, and concomitant increase in Complement component C7, Complement C4-B, Serum amyloid A-4 protein, Inter-α-trypsin inhibitor heavy chain H4, and α-1-antitrypsin, we believe that the biomarkers identified in this case study should be considered in priority in future studies. Further investigation with larger samples sizes will help to elucidate their role in monitoring NFOR and overtraining syndrome.

## Data Availability Statement

The datasets generated for this study are available on request to the corresponding author.

## Ethics Statement

The studies involving human participants were reviewed and approved by the Appalachian State University Institutional Review Board. The participants provided written informed consent to participate in this study and for the publication of any potentially identifiable images or data included in this article.

## Author Contributions

EM, BT, and DN designed the study. EM and BT collected the samples and data during RAAM. AG and AP analyzed the blood samples. All authors contributed to writing the manuscript, analyzed the data, and were responsible for revising the intellectual content of the manuscript, and reading and approving the final version of the manuscript.

## Conflict of Interest

AG and AP were employed by ProteiQ Biosciences GmbH. The remaining authors declare that the research was conducted in the absence of any commercial or financial relationships that could be construed as a potential conflict of interest.
